# Neurological Manifestations of Renal Diseases in Children in Qazvin/ Iran

**Published:** 2016

**Authors:** Reza DALIRANI, Abolfazl MAHYAR, Parviz AYAZI, Ghazaleh AHMADI

**Affiliations:** 1 Pediatric Nephrology Department, Mofid Children Hospital, ShahidBeheshti University of Medical Sciences, Tehran, Iran; 2 Pediatric Department, Qazvin Children Hospital, Qazvin University of Medical Sciences, Qazvin, Iran; 3 General Practitioner, Qazvin Children Hospital, Qazvin University of Medical Sciences, Qazvin, Iran

**Keywords:** Neurologic manifestations, Renal disease, Children

## Abstract

**Objective:**

Renal diseases are one of the most common causes of referrals and admissions of children, hence it is important to know their neurological presentations. This study aimed to determine neurological presentations of renal diseases in children.

**Material & Methods:**

A total of 634 children with renal diseases, admitted to Qazvin Pediatric Hospital, Qazvin, central Iran from 2011 to 2013 were studied. Neurological presentations of patients were established and the results were analyzed using statistical tests.

**Results:**

Neurological presentations were found in 18 (2.8%) out of 634 patients, of whom 15 had febrile seizures, two thromboembolism, and one encephalopathy. Among patients with urinary tract infection (UTI), 2.6% had febrile seizures, 11.1% of those with glomerulonephritis had encephalopathy, and 3.7% of those with nephrotic syndrome had cerebral thromboembolism.

**Conclusion:**

Results showed neurological presentations in 2.8% of children with renal diseases, and febrile seizure as the most common presentation.

## Introduction

Renal diseases are one of the most common causes of referrals and admissions of children. Common renal diseases include urinary tract infection (UTI), nephrotic syndrome and glomerulonephritis such as post streptococcal glomerulonephritis (PSGN). Urinary tract infection is one of the most common diseases in children. Delayed diagnosis and treatment can cause permanent complications such as renal scarring, hypertension, and renal failure. Nephrotic syndrome is another disease that can be associated with neurological complications. The prevalence of nephrotic syndrome is about 16/100,000 children per year (1). It is identified with triad of proteinuria, edema, and hypoalbuminemia. One of the major complications of nephrotic syndrome is thromboembolic event. PSGN is the most common form of glomerulonephritis in children characterized by the sudden onset of gross hematuria, edema, and hypertension following streptococcal infection (1). Common presentations of renal diseases are frequency, dysuria, edema, hematuria, and hypertension (1). Neurological symptoms are an important presentation in children with renal diseases (2, 3), which include febrile seizures, encephalopathy, and cerebral thromboembolism (2, 3). Some children withUTI present with febrile seizure (3). Given the importance of recognizing of uncommon symptoms of renal diseases, this study aimed to identify neurological presentations in children with renal diseases.

## Materials & Methods

In this descriptive cross sectional study, 634 children with renal diseases were evaluated in terms of frequency of neurological presentations. This study was conducted in Qazvin Children Hospital affiliated to Qazvin University of Medical Sciences, Qazvin, central Iran from 2011 to 2013. Sampling was conducted by census, and all patients with confirmed diagnosis of renal disease by pediatric nephrologists were studied. The inclusion criteria were the age between one month to 12 yr old and existence of renal disease. Diseases other than renal diseases were excluded. The Ethics Committee of the Research Department in Qazvin University of Medical Sciences approved this study. The following definitions were considered for renal diseases: Urinary tract infection was considered as a positive urine culture (urine culture more than105 colonies of a single pathogen in a midstream urine sample or clean catch method or 104 colonies of a single pathogen via urinary catheterization, or presence of any number of colonies of an organism in urine culture taken by suprapubic method) (4). Nephrotic syndrome was defined as generalized edema, massive proteinuria (>40 mg/m2 /h), hypoalbuminemia (250 mg/dl) and increase of triglyceride (5). Acute glomerulonephritis was considered as a pathological process that may be manifested clinically as an acute nephritic syndrome or rapidly progressive glomerulonephritis. Post streptococcal glomerulonephritis (PSGN) is a classic example of the acute nephrotic syndrome characterized by the sudden onset of gross hematuria, edema, hypertension, and renal insufficiency (6). Hemolytic uremic syndrome (HUS) was defined as a thrombotic microangiopathy characterized by three primary symptoms: hemolytic anemia with fragmentocytes, low platelet count and acute renal failure (7). Febrile seizure was defined as a type of seizureaccompanied by fever (t≥38o C) seen in children aged 6 months to 5 yr with no history of electrolyte imbalance, infection in central nervous and metabolic disorders. Simple febrile seizures are generalized, last for 15 min), focal, and recur within 24 h (8). Thrombosis was defined as intravascular blood coagulation that leads to thrombus formation. Embolism was defined as a portion of the thrombus that breaks free and flows downstream in the circulation and blocks flow to vital organs. Collectively these phenomena were considered as thromboembolism (9). The encephalopathy was defined as the reversible global change in brain function manifesting with attentional impairment, sleep-wake cycle disturbances, deficits in memory and mental data processing, and changes in arousal (hyper- or hypoactive) (10). First, information form was prepared, and then, demographic details, clinical symptoms including neurologic manifestations, and final diagnosis for all children with renal diseases were extracted and recorded. Data obtained were analyzed using Chi-squared test in SPSS-16 software (Chicago, IL, USA).

## Results

Of 22535 children admitted to Qods Children Hospital, 634 (2.8%) had renal diseases, of whom, 177 (27.8%) were admitted in 2011, 244 (38.7%) in 2012, and 213 (33.5%) in 2013. Of 634 children with renal diseases, 523 (82.4%) were female and 111 (17.6%) were male, the youngest was 1 month old, and the oldest 144 months old, with median ± IQR= 30±50 months. Of 634 children with renal diseases, 571 (89.8%) had urinary tract infections, 9 (1.4%) had glomerulonephritis (7 cases of PSGN, and 2 chronic cases), and 54 (8.5%) had nephrotic syndrome (mostly, minimal change). No hemolytic uremic syndrome cases were found. Of 634 patients, 18 (2.8%) had neurological presentations, of whom, 15 had febrile seizures, 2 had cerebral thromboembolism, and 1 encephalopathy. Of 571 patients with UTI, 15 (2.6%) had febrile seizures, of 54 patients with nephrotic syndrome, 2 (3.7%) had thromboembolism, and of 9 patients with glomerulonephritis, 1 (11.1%) had encephalopathy. All patients with clinical presentations of febrile seizurehad urinary tract infections. Febrile seizure was the first sign of urinary tract infection in these patients and cause of admission. The youngest and the oldest patients with febrile seizure were 7 months and 60 months, respectively, with median ± IQR= 27.7 ± 19.1. Of 15 patients with febrile seizures, 2 were male. All patients with febrile seizures had the simple febrile seizure. Of 15 children with UTI and febrile seizures, 10 were infected with E. coli, and 5 with other organisms. In 557 UTI cases without febrile seizures, 535 cases had E. coli, and 22 cases had other organisms (P=0.78). Two patients with cerebral thromboembolism suffered nephrotic syndrome, of whom, one was a 61 month-old male and one 133 month-old female, and both showed abnormal hemiparesis with MRI manifestation. One patient with clinical presentation of encephalopathy was a 60 month-old female with post streptococcal glomerulonephritis (PSGN), and her encephalopathy had been caused by hypertensive-encephalopathy.

## Discussion

The present study showed that 2.8% of children with renal diseases had neurological presentations and febrile seizure was the most common neurological presentation. Studies in this regard are infrequent. A study on 403 patients between 1 to 71 month-old children with febrile seizures showed that 7 (1.7%) of patients had UTI (3). Another study on 137 children between 1 to 144 months –old with febrile seizures showed that 6.6% of patients had UTI (11). Our results showed that the frequency of children with UTI suffered febrile seizures was higher than Lee et al. study (3) and lower than Momen et al. study (11). Considering that the incidence of renal scarring following UTI is more likely in young infants compared to other age groups (4), and that febrile seizure is also common in this age group (8), authors advise that any child with febrile seizure should be screened for UTI. Early diagnosis and rapid treatment in this age group can prevent renal complications such as renal scarring (1-4, 3, 11). In another study, 7% of children with glomerulonephritis showed clinical presentations of encephalopathy (12). In our study the frequency of encephalopathy manifestation was higher than the latter study. The causes of encephalopathy in glomerulonephritispatients are hypertension and uremic encephalopathy (1). The study of Brice et al. on 326 patients younger than 21 yr with nephrotic syndrome showed that the incidence of cerebral thromboembolism was 9.2% (9). The prevalence of thromboembolism in children with idiopathic nephrotic syndrome was 1.8% to 5% (13). The incidence of thromboembolism is much higher in adult patients (20% to 30%) (13, 14). Another study reported that about 2% to 5% of children with nephrotic syndrome suffer thromboembolism complications such as cerebral venous thrombosis (CVT) (15). In our study, the frequency of cerebral thromboembolism was lower than mentioned studies. Joel et al. believe that the etiology of thromboembolism is hypercoagulable state (16). This phenomenon is created by various mechanisms, including higher production of fibrinogens and coagulation factors, thrombocytosis, increased platelet adhesion, increased urinary excretion of antithrombin III, protein C, protein S, dehydration, diuretic use and inherited thrombophilia (16). Although other renal diseases such as hemolytic uremic syndrome, uremic encephalopathy and renal transplantation can produce neurological manifestations (12, 17) but in the present study none of these diseases were found. The difference in the prevalence of neurological presentations in our study and mentioned studies may be due to differences in study type and method, sample size, and hospital type (referrals or non-referrals). In the present study, a patient with PSGN had presented with impaired consciousness and cortical symptoms, and cerebral symptoms had been caused by increased blood pressure and hypertensive-encephalopathy. The results of our study showed that neurological manifestations of pediatric renal diseases still are common. Limitation of our study was the patients who have not referred to our hospital.

In conclusion, every patient with neurological manifestations should remind us regarding pediatric renal diseases and subsequently investigation of that.
